# Editorial: Vascular and skeletal crosstalk in health and disease

**DOI:** 10.3389/fendo.2022.1084940

**Published:** 2022-11-15

**Authors:** Kunal Sharan

**Affiliations:** ^1^ Department of Molecular Nutrition, CSIR-Central Food Technological Research Institute, Mysuru, India; ^2^ Academy of Scientific and Innovative Research (AcSIR), Ghaziabad, India

**Keywords:** skeleton, angiogenesis, vascular system, bone, osteoporosis

Skeleton is a highly vascularized metabolically active hard connective tissue. The vascular structure in the bone supplies nutrients and endocrine/paracrine growth factors for its development, regular remodeling, and regeneration (1). In addition, the vascular endothelial cells in the bone tissue regulates bone metabolism by the secretion of various chemokines and growth factors (2). In return, bone cells also secrete angiogenic factors to regulate vascularization (3). The disturbance between the delicate balance of the vascular system and skeleton may lead to various pathological conditions (2). Besides, many bone pathologies have been linked to changes in vasculature. Moreover, there is a positive correlation between low bone mass and arterial/aortic calcification (4).

This Research Topic covers the articles focusing on the crosstalk between the skeleton and vascular system to regulate each other’s functions. The collection contains four articles: One *in vitro* study, one observational cross-sectional study, one meta-analysis, and one review article.

## Role of interleukin 35 in the coupling of bone remodeling and angiogenesis

IL-35 is an immunosuppressive cytokine produced by T regulatory and B cells. In this issue, Zhang et al. investigated how IL-35 affects receptor activator of nuclear factor kappa-B ligand (RANKL) and macrophage colony-stimulating factor (MCSF) stimulated RAW cells’ differentiation towards osteoclast and their angiogenesis. Their results demonstrated that IL-35 promotes the apoptosis of RANKL and M-CSF-stimulated osteoclasts and inhibits the secretion of vascular endothelial growth factor (VEGF). The inhibition of the expression of VEGF and its receptor was found to be through the downregulation of the Th17/IL-17-related pathway. The results from the study show that IL-35 can be one of the factors connecting bone mass with angiogenesis. However, further validation of the results in animal models and detailed elucidation of the molecular mechanism is mandated.

## Effect of blood pressure on bone mineral density and fracture

Hypertension is often linked to stroke and coronary artery disease. Many previous studies linking high blood pressure with bone mineral density had conflicting outcomes. In this issue, He et al. performed a meta-analysis using Mendelian randomization (MR) to connect hypertension to bone mass. MR overcomes the limitations of confounding factors and reverses causation bias, which predominantly occurs in observational studies. The authors used single-nucleotide polymorphisms (SNPs) strongly associated with blood pressure to explore their effect on bone mineral density (BMD) and fractures at various sites.

The authors found a causal relationship between genetically high pulse pressure (PP) and improved forearm-BMD (FA-BMD). However, high PP is also associated with vascular calcification and arterial stiffness, often linked to increased osteoblast activity. Further investigations are required to delineate whether the improved BMD is associated with the altered function of the genes or increased blood pressure.

## Osteocalcin and aortic calcification

Jia et al. performed a cross-sectional observational study in hemodialysis patients with chronic kidney disease–mineral and bone disorder (CKD–MBD) to investigate the correlation between serum osteocalcin (OC) levels and vascular calcification. Vascular calcification is highly prevalent in both the osteoporotic and hemodialysis population. The authors demonstrated that elevated serum OC was a risk factor for abdominal aortic calcification. Although this study did not differentiate between carboxylated-OC and undercarboxylated-OC, it’s an important lead for future studies in this direction.

## Chronic obstructive pulmonary disease and muscle-bone communication

An interesting article by Zhang et al. reviewed the alteration in muscle-bone crosstalk in COPD patients. COPD is characterized by an obstruction in the airflow by the lungs due to perivascular inflammation. Low bone mass (osteopenia) and muscle wasting (sarcopenia) are two critical comorbidities of COPD. This review comprehensively describes how bone loss and muscle dysfunction processes are interconnected and regulate each other in the case of COPD. The authors propose that the pathogenesis of sarcopenia and osteoporosis in COPD are linked through myokines and osteokines released by muscle and bone, respectively.

## Conclusion

The current Research Topic highlights the impact of the coupling of the vascular and skeletal systems in regulating each other’s functions. The communication between the bone and vasculature is both ways and should be seen in conjunction. Besides, certain regulators, like cytokines and growth factors, modulate both bone and angiogenesis. The area is evolving, and more research is required to understand vascular and skeletal crosstalk better.

## Author contributions

The author listed have made a substantial, direct and intellectual contribution to the work, and approved it for publication.

## Conflict of interest

The author declares that the research was conducted in the absence of any commercial or financial relationships that could be construed as a potential conflict of interest.

## Publisher’s note

All claims expressed in this article are solely those of the authors and do not necessarily represent those of their affiliated organizations, or those of the publisher, the editors and the reviewers. Any product that may be evaluated in this article, or claim that may be made by its manufacturer, is not guaranteed or endorsed by the publisher.
